# Metabolic Interplay between Peroxisomes and Other Subcellular Organelles Including Mitochondria and the Endoplasmic Reticulum

**DOI:** 10.3389/fcell.2015.00083

**Published:** 2016-01-28

**Authors:** Ronald J. A. Wanders, Hans R. Waterham, Sacha Ferdinandusse

**Affiliations:** Laboratory Genetic Metabolic Diseases, Laboratory Division, Departments of Paediatrics and Clinical Chemistry, Academic Medical Center, Emma Children's Hospital, University of AmsterdamAmsterdam, Netherlands

**Keywords:** peroxisomes, mitochondria, endoplasmic reticulum, fatty acids, etherphospholipids, genetic diseases, peroxisomal diseases, metabolism

## Abstract

Peroxisomes are unique subcellular organelles which play an indispensable role in several key metabolic pathways which include: (1.) etherphospholipid biosynthesis; (2.) fatty acid beta-oxidation; (3.) bile acid synthesis; (4.) docosahexaenoic acid (DHA) synthesis; (5.) fatty acid alpha-oxidation; (6.) glyoxylate metabolism; (7.) amino acid degradation, and (8.) ROS/RNS metabolism. The importance of peroxisomes for human health and development is exemplified by the existence of a large number of inborn errors of peroxisome metabolism in which there is an impairment in one or more of the metabolic functions of peroxisomes. Although the clinical signs and symptoms of affected patients differ depending upon the enzyme which is deficient and the extent of the deficiency, the disorders involved are usually (very) severe diseases with neurological dysfunction and early death in many of them. With respect to the role of peroxisomes in metabolism it is clear that peroxisomes are dependent on the functional interplay with other subcellular organelles to sustain their role in metabolism. Indeed, whereas mitochondria can oxidize fatty acids all the way to CO_2_ and H_2_O, peroxisomes are only able to chain-shorten fatty acids and the end products of peroxisomal beta-oxidation need to be shuttled to mitochondria for full oxidation to CO_2_ and H_2_O. Furthermore, NADH is generated during beta-oxidation in peroxisomes and beta-oxidation can only continue if peroxisomes are equipped with a mechanism to reoxidize NADH back to NAD^+^, which is now known to be mediated by specific NAD(H)-redox shuttles. In this paper we describe the current state of knowledge about the functional interplay between peroxisomes and other subcellular compartments notably the mitochondria and endoplasmic reticulum for each of the metabolic pathways in which peroxisomes are involved.

## Introduction

Eukaryotic cells contain a variety of different subcellular compartments, which differ from one another in multiple aspects including their biogenesis, enzyme content, and role in metabolism. Lysosomes, for instance, are primarily involved in the breakdown of macromolecules, whereas mitochondria are the ultimate site of aerobic metabolism. Peroxisomes perform both catabolic as well as anabolic functions. The role of peroxisomes in human metabolism and their importance for human health and development has largely been elucidated thanks to the detailed work on a rare genetic human disease called the cerebro-hepato-renal syndrome, or Zellweger syndrome (ZS). In patients with the classical form of ZS, functional peroxisomes are lacking due to a genetically determined defect in the biogenesis of peroxisomes. ZS is genetically heterogeneous, which is explained by the fact that peroxisome biogenesis involves the obligatory participation of multiple proteins required for the formation, proliferation, and maintenance of these organelles (Mast et al., 2010; Waterham and Ebberink, 2012; Hasan et al., 2013; Fujiki et al., 2014). The proteins involved are called peroxins and are encoded by *PEX* genes. Clinically, ZS patients show multiple abnormalities including cranial facial, neurological, hepatic, and other aberrations, and usually die early in life. Work on ZS has resulted in the discovery of the various unique metabolic functions exerted by peroxisomes which include: (A) etherphospholipid biosynthesis; (B) fatty acid beta-oxidation; (C) docosahexaenoic acid synthesis; (D) bile acid synthesis; (E) fatty acid alpha-oxidation; (F) glyoxylate detoxification; (G) amino acid metabolism, and (H) ROS/RNS-metabolism.

The essential role of peroxisomes in each of these metabolic pathways is emphasized by the fact that a variety of genetic diseases in man has been identified, usually with severe clinical signs and symptoms, that are caused by mutations in genes coding for peroxisomal enzymes involved in each of these metabolic pathways. Table 1 lists the single peroxisomal enzyme deficiencies identified so far including two recently identified peroxisomal disorders including fatty acyl-CoA reductase 1 (FAR1) deficiency (Buchert et al., 2014) and Peroxisomal Membrane Protein 70 (PMP70/ABCD3) deficiency (Ferdinandusse et al., 2015).

**Table 1:** **The single peroxisomal enzyme deficiencies and their underlying enzyme and gene defects**.

**Metabolic pathway involved**	**Peroxisomal disorders**	**Enzyme deficiencies**	**Mutant gene**
**PEROXISOMAL BETA-OXIDATION**			
	• X-linked adrenoleukodystrophy	ALDP	*ABCD1*
	• Acyl-CoA oxidase deficiency	ACOX1	*ACOX1*
	• D-bifunctional protein deficiency	DBP, MFE2, MFP2, D-PBE	*HSD17B4*
	• SCPx-deficiency	SCPx	*SCP2*
	• AMACR deficiency	AMACR	*AMACR*
	• PMP70-deficiency	PMP70	*ABCD3*
**PLASMALOGEN BIOSYNTHESIS**			
	• RCDP Type 2	DHAPAT/GNPAT	*GNPAT*
	• RCDP Type 3	ADHAPS/AGPS	*AGPS*
	• RCDP Type 4	Acyl-CoA reductase 1	*FAR1*
	• RCDP Type 5	PEX5L	*PEX5*
**FATTY ACID ALPHA-OXIDATION**			
	• Refsum disease	Phytanoyl-CoA hydroxylase	*PHYH*
**BILE ACID SYNTHESIS**			
	• BAAT-deficiency	BAAT	*BAAT*
**GLYOXYLATE METABOLISM**			
	• Hyperoxaluria Type 1	AGT	*AGXT*
**ROS/RNS METABOLISM**			
	• Acatalasaemia	Catalase	*CAT*

**Figure 1:**
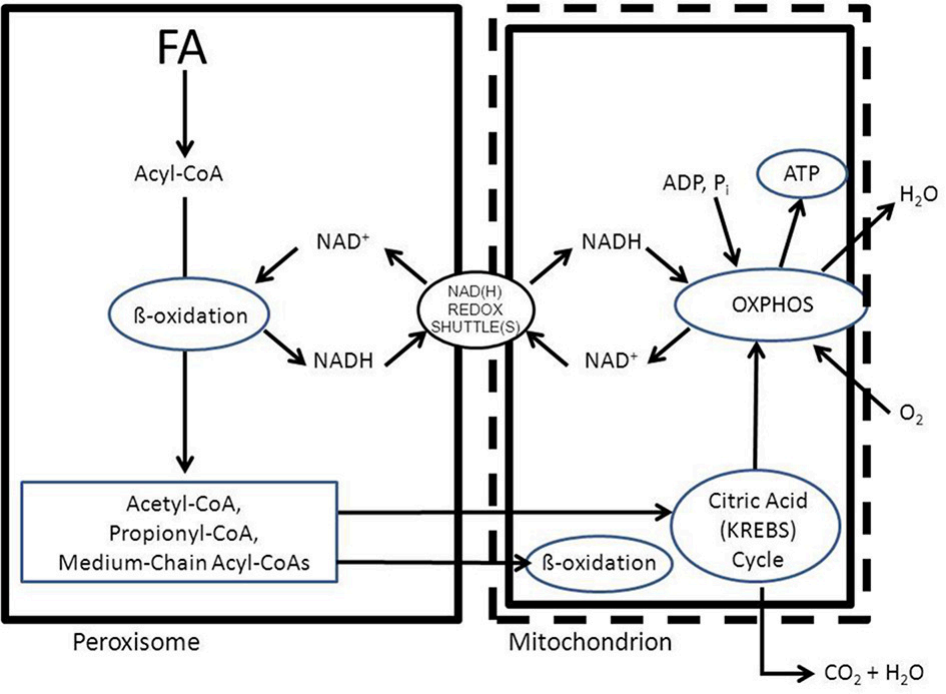
**Schematic diagram showing the functional interplay between peroxisomes and mitochondria in the beta-oxidation of fatty acids in peroxisomes**. See text for detailed discussion.

To fulfill their role in metabolism peroxisomes rely very much on the interaction with other subcellular organelles. For instance, beta-oxidation of fatty acids (FAs) in peroxisomes generates NADH from NAD^+^. Reoxidation of NADH back to NAD^+^, however, relies on the interaction with the cytosol and subsequently the mitochondrion (see Figure 1). Furthermore, peroxisomes produce a range of chain-shortened acyl-CoAs including acetyl-CoA, which can only be fully oxidized to CO_2_ and H_2_O in mitochondria and not in peroxisomes. This is due to the fact that mitochondria contain the citric acid (KREBS) cycle, which catalyses the degradation of acetyl-CoA to CO_2_ and H_2_O (Figure 1). Also for other metabolic functions, peroxisomes depend on the interaction with other subcellular organelles. In this paper we will describe the interactions between peroxisomes and other subcellular organelles for each of the metabolic functions of peroxisomes in humans.

### (A) etherphospholipid biosynthesis

**Figure 2:**
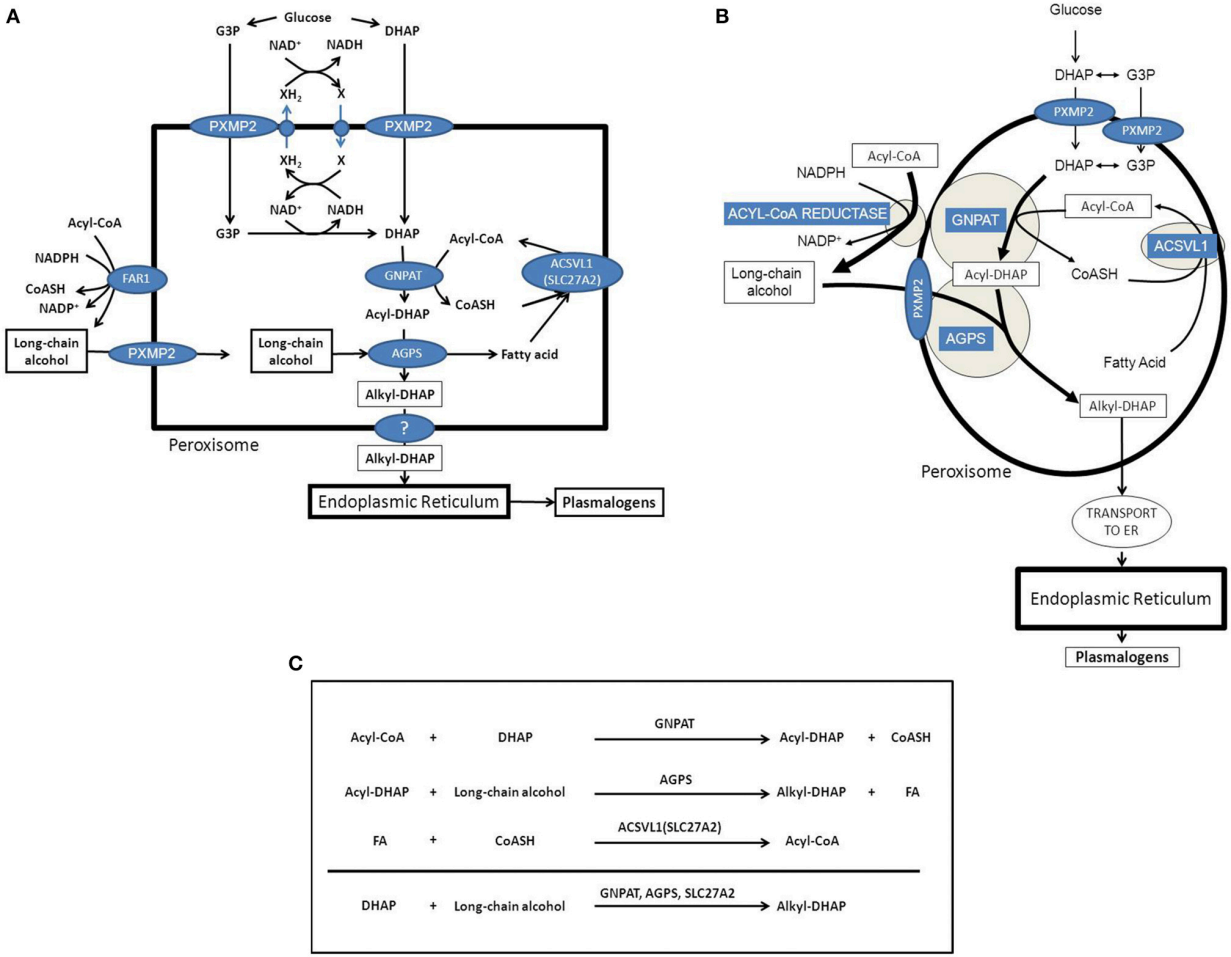
**Etherphospolipid biosynthesis and the important role of peroxisomes and the endoplasmic reticulum**. **(A)** Schematic representation of the peroxisome and the enzymes and transporters involved in the peroxisomal synthesis of alkyl-DHAP. **(B)** Schematic representation of the peroxisome and the enzymes and transporters involved in the peroxisomal synthesis of alkyl-DHAP with special emphasis on the sub-peroxisomal localization of GNPAT and AGPS as peripheral peroxisomal membrane proteins joined together in a functional complex. **(C)** Overview of the reactions catalyzed by the three intraperoxisomal enzymes involved in etherphospholipid synthesis including GNPAT, AGPS, and ACSVL1 (SLC27A2). G3P, glycerol-3-phosphate; DHAP, dihydroxyacetonephosphate; GNPAT, glyceronephosphate O-acyltransferase; AGPS, alkyl-glyceronephosphate synthase; FAR1, fatty acyl-CoA reductase 1.

The first major function of peroxisomes in metabolism involves the synthesis of ether-linked phospholipids (EPLs). In humans most EPLs occur in their plasmalogen form (Brites et al., 2004). Peroxisomes are indispensable for etherphospholipid synthesis, which is due to the fact that the enzyme responsible for the generation of the ether-bond in EPLs is strictly peroxisomal. The enzyme involved is alkyl-glyceronephosphate synthase [AGPS; formally named alkyldihydroxyacetonephosphate synthase (ADHAPS)] which catalyses the exchange of the acyl-group in acyl-DHAP for a long-chain alcohol via an ingenious mechanism to ensure formation of the ether-bond. The two substrates for AGPS include acyl-DHAP and a long-chain alcohol which are both synthesized by peroxisomal enzymes including glycerone-phosphate O-acyltransferase [GNPAT; formally called dihydroxyacetonephosphate acyltransferase (DHAPAT)] and one of two peroxisomal acyl-CoA reductases named FAR1 and FAR2 (Cheng and Russell, 2004). Both AGPS/ADHAPS as well as GNPAT/DHAPAT are true intra-peroxisomal enzymes targeted to peroxisomes via the PTS1- and PTS2-import pathways respectively (Mast et al., 2010; Waterham and Ebberink, 2012; Hasan et al., 2013; Fujiki et al., 2014; Figure 2A). Both enzymes are bound to the inner site of the peroxisomal membrane and form a functional complex (see Figure 2B). On the other hand, earlier work by Hajra and coworkers (Burdett et al., 1991) had shown that the situation is different with respect to the third peroxisomal enzyme involved in etherphospholipid biosynthesis which is the enzyme acyl-CoA reductase. This enzyme catalyzes the synthesis of the long-chain alcohol from the corresponding acyl-CoA ester using NADPH as reductant (Bishop and Hajra, 1981). The enzyme from rat brain (Bishop and Hajra, 1981) showed high specificity for palmitoyl-CoA (C16:0-CoA), stearoyl-CoA (C18:0-CoA), and oleoyl-CoA (C18:1-CoA). Importantly, this acyl-CoA reductase activity was found at the outer aspect of the peroxisomal membrane, which implies that the substrates including the acyl-CoA ester plus NADPH are in the cytosol and not localized in the peroxisomal matrix (see Figure 2). Most likely, the long-chain alcohol produced in the acyl-CoA reductase reaction is also released at the cytosolic face of the peroxisomal membrane (see Figure 2B; Honsho et al., 2013).

Work by the group of Russell has led to the identification and characterization of two acyl-CoA reductase isozymes named FAR1 and FAR2 (Cheng and Russell, 2004). The two FAR isozymes are ~58% identical in sequence and are encoded by two distinct genes with similar exon/intron structures located on different chromosomes. FAR1 has a broad tissue distribution and acts on acyl-CoAs that vary in size and saturation suggesting that this isozyme plays a general role in the synthesis of fatty alcohols. In contrast, the more narrow tissue distribution and substrate preference of the FAR2 isozyme are indicative of a more specialized function. Recent work by Fujiki and coworkers (Honsho et al., 2010, 2013) has revealed that FAR1 is a peroxisomal tail-anchored protein targeted to peroxisomes via PEX19 (Honsho et al., 2013). Furthermore, the expression of FAR1 but not FAR2 is posttranslationally regulated. Indeed, FAR1 but not FAR2 is preferentially degraded in response to the cellular level of plasmalogens (Honsho et al., 2010, 2013). Peroxisomes have also been found to contain alkyl-DHAP reductase activity although the bulk of activity is in the endoplasmic reticulum.

The indispensable role of the peroxisomal enzymes GNPAT (Wanders et al., 1992), AGPS (Wanders et al., 1994), and FAR1 (Buchert et al., 2014) in the formation of etherphospholipids has become clear from the identification of patients with genetically determined deficiencies of these three enzymes. In such patients EPL-synthesis is severely deficient as concluded from the markedly reduced plasmalogen levels in erythrocytes from patients (for reviews see Brites et al., 2004; Braverman and Moser, 2012; Malheiro et al., 2015).

#### Etherphospholipid biosynthesis and the interplay with other organelles

GNPAT and AGPS are true intraperoxisomal enzymes targeted to peroxisomes via the PTS1- and PTS2-pathways respectively. Inspection of the two reactions catalyzed by GNPAT and AGPS reveals that the acyl-CoA ester used by GNPAT to produce acyl-DHAP, is released in its free acid form in the AGPS-catalyzed reaction, whereas the CoA unit is released in the GNPAT catalyzed reaction. Taken together it would make sense to regenerate the acyl-CoA ester within the peroxisome via an acyl-CoA synthetase. Peroxisomes contain at least one truly intraperoxisomal acyl-CoA synthetase called ACSVL1 (SLC27A2), which is a peripheral membrane protein facing the interior of the peroxisome. The same enzyme also occurs in the endoplasmic reticulum (ER; Steinberg et al., 1999). If such a scenario would be true, the formation of alkyl-DHAP would be as depicted in Figure 2B, the net equation being: DHAP + long-chain alcohol → alkyl-DHAP (see Figure 2C). The implication of this would be that DHAP and the long-chain alcohol need to be transported from outside the peroxisome, whereas alkyl-DHAP needs to be exported from the inside of the peroxisome to the outside. DHAP synthesized from glucose in the cytosol can move into the peroxisome interior without any problem if it is true that small solutes up to an M_w_of 300 Da, can move across the peroxisomal membrane freely via PXMP2 (Rokka et al., 2009). However, it has also been claimed that DHAP is synthesized endogenously from glycerol-3-phosphate (G3P) as catalyzed by the enzyme glycerol-3-phosphate dehydrogenase (G3PDH/GPD1). Interestingly, earlier work from Antonenkov (Antonenkov et al., 1985) has shown that rat liver peroxisomes do contain G3PDH activity although the true identity of this enzyme activity has never been resolved definitively. In this respect it is important to mention the work of Jung et al. in yeast which revealed dynamic changes in the subcellular distribution of G3PDH ranging from peroxisomal to cytosolic depending on the metabolic status of the cells (Jung et al., 2010). Either way, both G3P and DHAP would qualify for transport across the peroxisomal membrane via the peroxisomal porine PXMP2 (Rokka et al., 2009).

Since the contribution of peroxisomes to EPL-biosynthesis is restricted to the formation of alkyl-DHAP, or—at best—alkyl-G3P, completion of EPL-biosynthesis is very much dependent upon the interaction with the rest of the cell. In fact, all subsequent steps in EPL-biosynthesis are catalyzed by ER-bound enzymes (see Figure 2).

It remains to be established whether the transfer of alkyl-DHAP and/or alkyl-G3P occurs via the cytosol with the possible involvement of a binding protein analagous to the binding of acyl-CoAs by acyl-CoA binding protein (ACBP), or whether this is mediated via direct interorganellar contact sites between peroxisomes and the endoplasmic reticulum. Ultrastructural studies in the early 1960s had already demonstrated a close proximity between the smooth ER and peroxisomes (Novikoff and Shin, 1964). Indeed, transmission electron microscopy analysis revealed that both organelles appear to be interconnected by electron-dense intermembrane cross-bridges spanning some 10–15 nm. Additional evidence in favor of the existence of such cross-bridges came from biochemical studies in which peroxisomes were isolated from bovine kidney (Zaar et al., 1987). The identity of the proteins mediating the physical interaction between peroxisomes and the ER remains to be identified.

### (B) fatty acid beta-oxidation

Like mitochondria, peroxisomes contain a fatty acid beta-oxidation machinery, which catalyses the stepwise shortening of acyl-CoAs to produce acetyl-CoA in case of straight-chain acyl-CoAs and propionyl-CoA when a 2-methyl-branched-chain acyl-CoA is oxidized. Although the beta-oxidation systems in peroxisomes and mitochondria are basically identical in chemical terms and involve four sequential steps of dehydrogenation, hydration, dehydrogenation again, and thiolytic cleavage, there are major differences between the two systems which include: (a.) the four reactions of the mitochondrial and peroxisomal beta-oxidation pathways are catalyzed by different enzymes each encoded by a distinct gene; (b.) the mitochondrial enzymes catalyzing the first step of beta-oxidation are FAD-dependent dehydrogenases, which feed their electrons into the respiratory chain via the Electron-Transfer-Flavoprotein (ETF) cycle, whereas the corresponding peroxisomal enzymes are FAD-dependent acyl-CoA oxidases donating their electrons directly to molecular oxygen (O_2_); (c.) fatty acids are transported across the peroxisomal membrane as acyl-CoAs, or as free fatty acids, whereas fatty acids are transported across the mitochondrial membrane as acylcarnitine esters mediated by the carnitine cycle via the concerted action of carnitine palmitoyl transferase 1 (CPT1), carnitine acylcarnitine translocase (CACT), and carnitine palmitoyl transferase 2 (CPT2); (d.) carnitine does not play a role in the uptake of fatty acids into peroxisomes but is required for the transport of the end products of peroxisomal beta-oxidation to mitochondria for full oxidation to CO_2_ and H_2_O, which requires the active participation of the citric acid cycle and the mitochondrial oxidative phosphorylation system (respiratory chain); (e.) mitochondria are able to degrade FAs to CO_2_ and H_2_O, whereas peroxisomes can only chain-shorten fatty acids to acetyl-CoA, propionyl-CoA, and different medium-chain acyl-CoAs, which all need to be transferred to mitochondria for full oxidation to CO_2_ and H_2_O, and (f.) both peroxisomes and mitochondria are equipped with auxiliary enzymes for the oxidation of unsaturated fatty acids and *2R*-methyl branched chain FAs.

**Figure 3:**
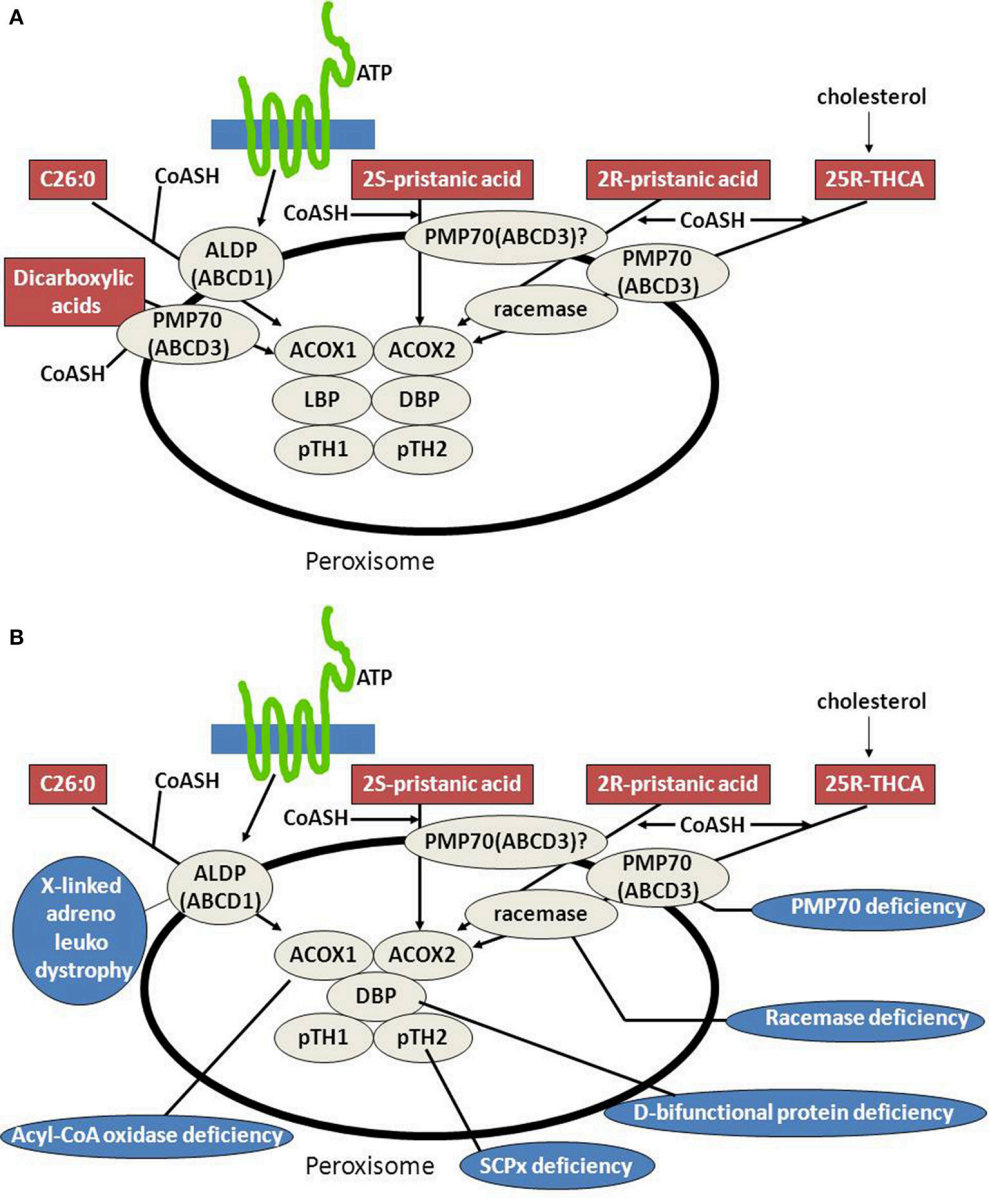
**Peroxisomes, fatty acid beta-oxidation and the human deficiencies of peroxisomal beta-oxidation. (A)** Schematic diagram depicting the substrates known to be oxidized in peroxisomes exclusively and the transporters and enzymes involved in their degradation (see text for detailed information). **(B)** Schematic diagram depicting the substrates known to be oxidized in peroxisomes exclusively and the transporters and enzymes involved in their degradation and the human deficiencies in the peroxisomal beta-oxidation pathway so far identified (see text for more information).

**Figure 4:**
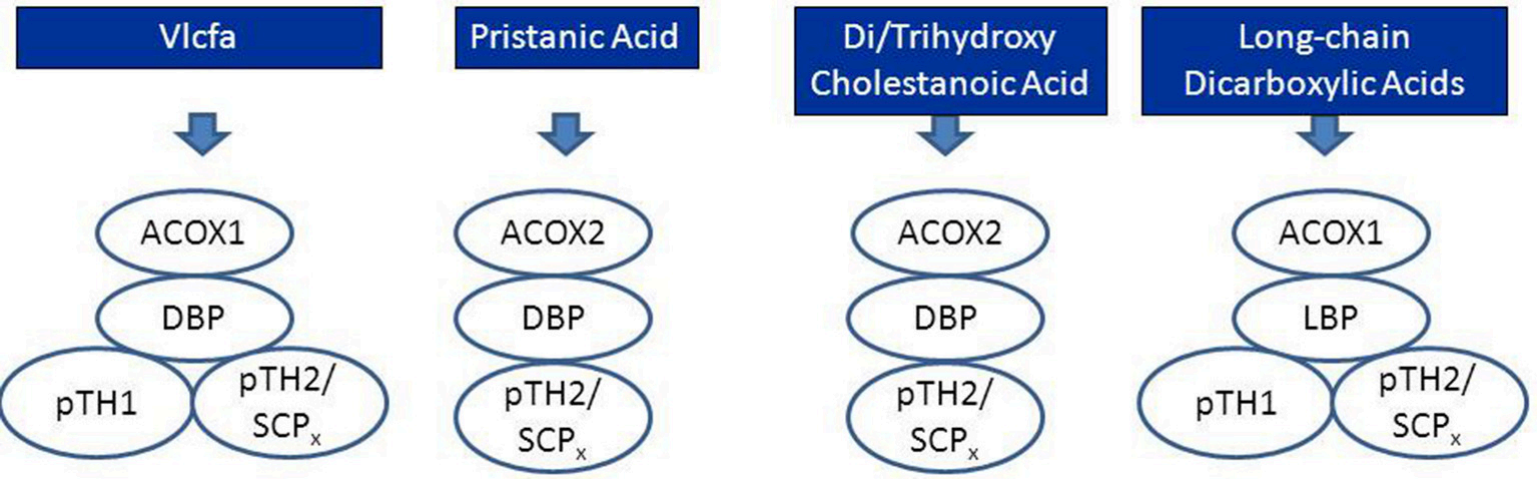
**Schematic diagram depicting the peroxisomal enzymes involved in the degradation of very long-chain fatty acids (VLCFAs), pristanic acid, di- and trihydroxycholestanoic acid, and long-chain dicarboxylic acids**. See text for detailed information.

Peroxisomal beta-oxidation of the various acyl-CoA esters is mediated by two acyl-CoA oxidases, two bifunctional proteins, and two thiolases (Figure 3A). Much of our knowledge about the physiological roles of the different enzymes involved has come from studies in human patients in whom one of the peroxisomal beta-oxidation enzymes is deficient due to mutations in the structural genes encoding these proteins (see Figure 3B, Table 1) as well as in mutant mice. Taken all data together, current knowledge holds that acyl-CoA oxidase 1 (ACOX1) is the main enzyme involved in the oxidation of VLCFAs and dicarboxylic acids (DCAs), whereas ACOX2, also called branched-chain acyl-CoA oxidase (BCOX), is the prime oxidase handling the CoA esters of pristanic acid and di- and trihydroxycholestanoic acid (DHCA/THCA) (Vanhove et al., 1993). The second and third steps of beta-oxidation in peroxisomes are catalyzed by two multifunctional proteins alternatively named L- and D-bifunctional protein (LBP and DBP), peroxisomal multifunctional enzyme type 1 and 2 (MFE1 and MFE2), multifunctional protein 1 and 2 (MFP1 and MFP2), and L- and D-peroxisomal bifunctional enzyme (L-PBE and D-PBE) which have been purified, characterized and cloned from various sources (Dieuaide et al., 1996; Leenders et al., 1996; Dieuaide-Noubhani et al., 1997; Jiang et al., 1997; Qin et al., 1997a,b). With respect to their physiological role, it is now clear that the D-specific enzyme as encoded by *HSD17B4* catalyzes the hydration and subsequent dehydrogenation of most peroxisomal beta-oxidation substrates including the enoyl-CoA esters of VLCFAs, pristanic acid and DHCA and THCA, whereas the L-specific enzyme appears to handle the dicarboxylic enoyl-CoA esters specifically (see Figure 4; Houten et al., 2012). Finally, with respect to the two thiolases, work on the isolated enzymes (Antonenkov et al., 1997; Wanders et al., 1997) as well as Sterol Carrier Protein X (SCPx)-deficient patients (Ferdinandusse et al., 2006) and mutant mice (Seedorf et al., 1998; Kannenberg et al., 1999) has shown that both peroxisomal 3-ketoacyl-CoA-thiolase 1 (ACAA1; pTH1) and SCPx (pTH2) encoded by *ACAA1* and *SCP2*, respectively, are involved in the oxidation of VLCFAs, whereas the 3-ketoacyl-CoA esters of pristanic acid, DHCA and THCA are solely cleaved by SCPx. The redundancy of the two thiolases with respect the oxidation of VLCFAs may explain why peroxisomal 3-ketoacyl-CoA-thiolase1 (ACAA1)-deficiency has not been identified sofar.

Peroxisomes are not only able to oxidize saturated FAs but also catalyze the oxidation of certain mono- and polyunsaturated FAs as well as 2-(*R*)-methyl branched-chain FAs and 2-hydroxy-FAs (see Wanders and Waterham, 2006; Van Veldhoven, 2010 for reviews). To this end, peroxisomes contain a set of so-called auxiliary enzymes including enoyl-CoA isomerase(s) and 2,4-dienoyl-CoA reductase(s) to remove the double bond present in mono- and polyunsaturated acyl-CoAs, respectively. It should be noted that it is currently unknown which mono- and/or polyunsaturated FAs (MUFA/PUFA) are solely oxidized in peroxisomes except from tetracosahexaenoic acid (C24:6 n-3) as discussed under (C.).

The peroxisomal branched-chain acyl-CoA oxidase ACOX2 only reacts with 2-methyl branched-chain acyl-CoAs if the methyl-group is in the 2(*S*)-position (Van Veldhoven et al., 1996). For DHCA and THCA, which are produced from cholesterol in the liver, this is a potential problem because the methyl-group in the carboxy-terminal side chain of DHCA and THCA has the 2(*R*)- rather than 2(*S*)- configuration which thus prohibits beta-oxidation. This problem is resolved by an enzyme called 2-methyl-acyl-CoA racemase (AMACR), located both in peroxisomes and mitochondria, which is able to convert 2(*R*)-acyl-CoAs into 2(*S*)-acyl-CoAs (Schmitz et al., 1997; Amery et al., 2000; Ferdinandusse et al., 2000; Kotti et al., 2000). The same enzyme is also required to convert the 2(*R*)-methyl-group in pristanic acid (2,10,14,16-tetramethylpentadecanoic acid) as derived from phytanic acid into the 2(*S*)-position (Figure 3A).

#### Peroxisomal beta-oxidation and the interplay with mitochondria

As already eluded to before, peroxisomes are dependent on other organelles for several of their metabolic functions. The interplay with mitochondria is especially important for further metabolism of the end-products of beta-oxidation in peroxisomes including: (1.) NADH; (2.) acetyl-CoA; (3.) propionyl-CoA, and (4.) a variety of acyl-CoAs chain-shortened in peroxisomes.

**Figure 5:**
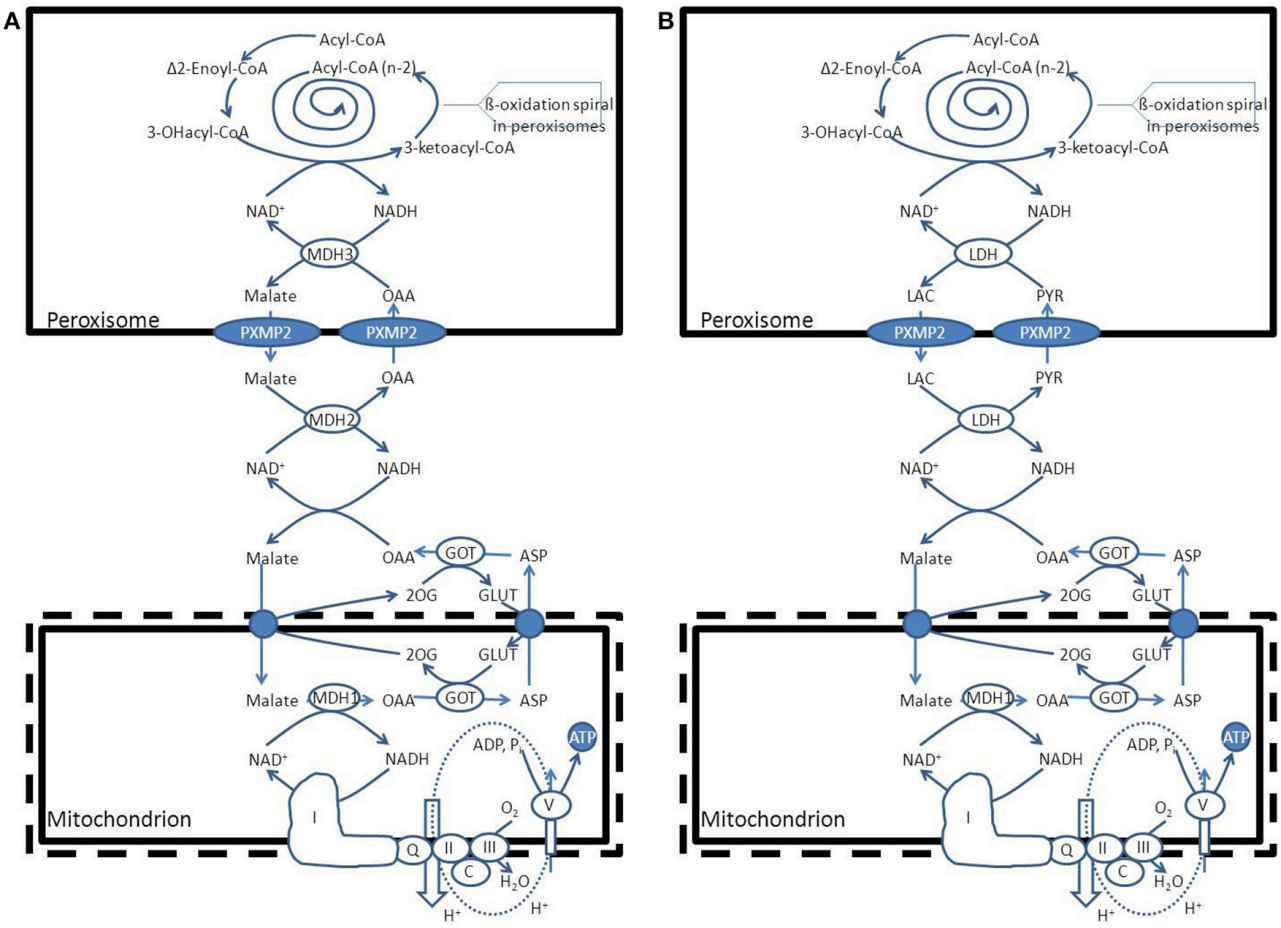
**Functional interplay between peroxisomes and mitochondria with respect to the reoxidation of NADH produced within the peroxisomal beta-oxidation system**. **(A)** *S. cerevisiae* (see text for detailed information). **(B)**: *H. sapiens*. PYR, pyruvate; LAC, lactic acid; LDH, lactate dehydrogenase; GOT, glutamate oxaloacetate transaminase; ASP, L-aspartate, GLUT, L-glutamate; 2OG, 2-oxoglutarate; MDH1, mitochondrial malate dehydrogenase.

**Figure 6:**
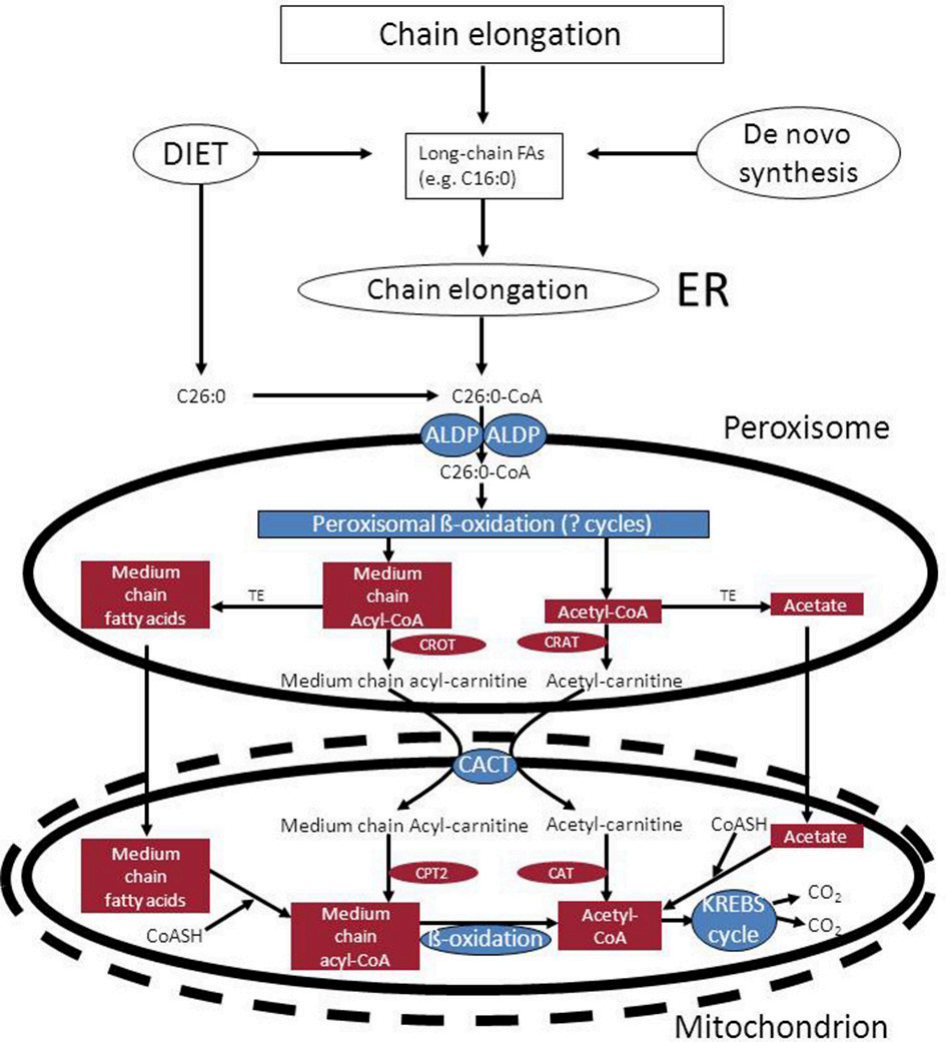
**Functional interplay between peroxisomes and mitochondria in the oxidation of C26:0**. See text for detailed information. ALDP, adrenoleukodystrophy protein; TE, acyl-CoA thioesterase; CrOT, carnitine octanoyltransferase; CrAT, carnitine acetyltransferase; CACT, mitochondrial carnitine: acylcarnitine translocase.

- *NADH reoxidation*: beta-oxidation in peroxisomes can only continue if the NADH formed in peroxisomes is reoxidized to NAD^+^. Ideally, a carrier system in the peroxisomal membrane catalyzing the exchange between NADH in the peroxisome and NAD^+^ in the cytosol would serve this purpose. However, since such a system appears to be lacking at least in higher eukaryotes, the existence of metabolite-based redox shuttles has been proposed analogous to the well-known malate/aspartate shuttle in mitochondria (see Figure 1). In baker's yeast (*S. cerevisiae*) the existence of a peroxisomal NAD(H)-redox shuttle has been demonstrated and involves a malate/oxaloacetate based redox shuttle for reoxidation of peroxisomal NADH (Figure 5A). In higher eukaryotes, however, including humans, the identity of the peroxisomal NAD(H)-redox shuttle has not been resolved definitively although evidence in favor of the existence of a lactate/pyruvate-based redox shuttle has been provided by Baumgart et al. (1996), at least in rat liver peroxisomes (see Figure 5B). Whatever the precise identity of the peroxisomal NAD(H)-redox shuttle, ultimate reoxidation of peroxisomal NADH back into NAD^+^, can only be achieved in mitochondria. The same is true for the NADH produced in the cytosol from glucose upon its conversion into pyruvate during glycolysis. Reoxidation of cytosolic NADH is mediated by one of two NAD(H)-redox shuttles including the malate/aspartate and glycerol-3-phosphate/dihydroxyacetonephosphate redox cycles.
- *Reduction of peroxisomal NADP back into NADPH*: as for the reoxidation of peroxisomal NADH, redox shuttles have been proposed also for the reduction of NADP as produced in the dienoyl-CoA reductase reaction back into NADPH. In the yeast *S. cerevisiae* there is strong evidence in favor of the existence of a peroxisomal 2-oxoglutarate/(iso) citrate NADP(H)-redox shuttle (van Roermund et al., 1998) next to a similar shuttle identified earlier in mitochondria. These two shuttle systems require the active participation of NADP-linked isocitrate dehydrogenases (IDHs) in mitochondria, peroxisomes and the cytosol respectively. These three activities are catalyzed by three distinct enzymes each encoded by a different gene, at least in *S. cerevisiae*. In higher eukaryotes, however, it appears that there are only two genes coding for NADP-linked IDHs, one in mitochondria, and the other one in peroxisomes, and the cytosol. Detailed work by Yoshihara and coworkers has shown that the bulk of the peroxisomal/cytosolic IDH-activity is actually peroxisomal (>90%), at least in hepatocytes (Yoshihara et al., 2001). As shown by Geisbrecht and Gould, the peroxisomal/cytosolic NADP-linked IDH is equipped with a true PTS1 sequence (Geisbrecht et al., 1999).
- *Mitochondria as ultimate site of oxidation of the different acyl-CoA esters produced in peroxisomes*: the acetyl-CoA, propionyl-CoA, and medium-chain acyl-CoAs produced in peroxisomes, ultimately require the mitochondrial citric acid cycle and oxidative phosphorylation system for full oxidation to CO_2_ and H_2_O. Shuttling of the different CoA-esters can occur via two mechanisms including: (1.) the carnitine-mediated pathway, and (2.) the free-acid pathway. The first pathway requires conversion of the different acyl-CoA species into acylcarnitines in peroxisomes. To this end, peroxisomes contain two distinct carnitine-acyltransferases, named carnitine-acetyltransferase (CrAT) and carnitine-octanoyltransferase (CrOT) reactive with short and medium-chain acyl-CoAs respectively. The different acylcarnitines are then exported out of the peroxisome via some unknown mechanism, and enter the mitochondrion via the mitochondrial carnitine/acylcarnitine translocase (CACT) followed by the reconversion of the acylcarnitines esters back into the corresponding acyl-CoAs followed by further degradation to CO_2_ and H_2_O. The alternative, free-acid route first involves cleavage of the acyl-CoA esters by one of a variety of different thioesterases that have been identified in peroxisomes (see Hunt et al., 2012 for review). Next, the free acids move out of the peroxisome probably through the porine (PXMP2) identified by Hiltunen and coworkers (see Rokka et al., 2009; Antonenkov and Hiltunen, 2012 for review) and then enter the mitochondrion most likely in their protonated form. Mitochondria contain both short-chain as well as medium-chain acyl-CoA synthetase activities to reactivate the free acids back into the corresponding acyl-CoAs followed by oxidation either directly (acetyl-CoA) or indirectly (propionyl-CoA and medium-chain acyl-CoAs). Figure 6 shows how this works out for C26:0. It remains to be established whether the transfer of metabolites from peroxisomes to mitochondria occurs via simple diffusion through the cytosol, or whether this is mediated through direct interorganellar contacts between peroxisomes and mitochondria. Close proximity between peroxisomes and mitochondria has been observed in intrastructural studies already many years ago (Hicks and Fahimi, 1977). In addition, there is biochemical evidence for direct peroxisome-mitochondrion interactions from density gradient centrifugation analyses (Islinger et al., 2006). Very recently, a genome-wide localization study of peroxisome-mitochondria interactions in yeast has led to the identification of a direct interaction between Pex11, a membrane-bound peroxin involved in peroxisome division and proliferation, and the mitochondrial ERMES complex. Interestingly, PEX11 was found to physically interact with Mdm34 to establish the contact between peroxisomes and mitochondria (see Schrader et al., 2015 for recent review). Tethering of both organelles is supposed to enhance metabolism by reducing the distance for efficient transport of metabolites from the peroxisome to the mitochondrion. Mammalian cells, however, lack ERMES so that another tethering complex is supposed to perform a similar function in higher eukaryotes, including humans.

### (C) docosahexaenoic acid (C22:6 n-3) synthesis

**Figure 7:**
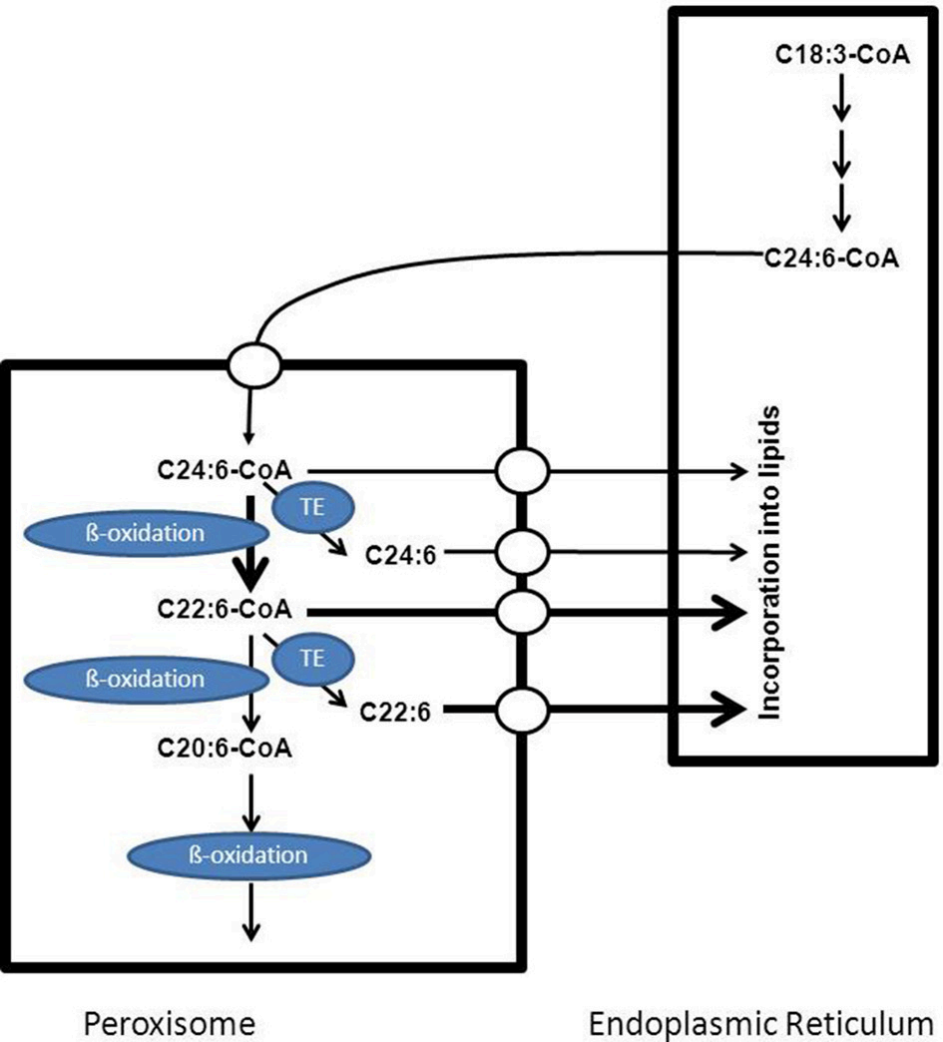
**Schematic diagram showing the key role of peroxisomes in the formation of C22:6 n-3**. Doxosahexaenoic acid is synthesized from C18:3 n-3 which first undergoes a number of elongation and desaturation steps in the endoplasmic reticulum to produce C24:6-CoA which is then transported to the peroxisome and imported via a mechanism not yet resolved. Within peroxisomes C24:6-CoA undergoes one cycle of beta-oxidation to produce the corresponding C22:6-CoA which can then be exported out of the peroxisome for subsequent incorporation into lipids in the endoplasmic reticulum or may undergo additional sequential rounds of oxidation in peroxisomes and mitochondria. See text for more detailed information.

DHA (C22:6 n-3) is the most important n-3 PUFA and is the major PUFA in adult mammalian brain and retina. A deficiency of DHA can lead to memory loss, learning disabilities and impaired visual acuity (Jump, 2002). DHA is synthesized from dietary linolenic acid (C18:3 n-3) in the endoplasmic reticulum via a series of elongation and desaturation reactions. This pathway requires that C22:5 n-3 would be desaturated at position 4 by an acyl-CoA-dependent delta4-desaturase to form C22:6 n-3. Several studies have shown that mammals do not possess such a delta4-desaturase. Instead, a 24-carbon n-3 fatty acid is first synthesized which is then desaturated at position six to produce C24:6 n-3 followed by one round of beta-oxidation in the peroxisome with C22:6 n-3 as final product (see Figure 7). Interestingly, some of the enzymes involved in the beta-oxidation of C24:6 n-3-CoA have been identified and include the straight-chain acyl-CoA oxidase (ACOX1) and D-bifunctional protein (Su et al., 2001; Ferdinandusse et al., 2003). The C22:6-CoA produced in peroxisomes may either undergo continued beta-oxidation in peroxisomes and subsequently in mitochondria or be exported out of the peroxisome for incorporation into lipids in the endoplasmic reticulum. The exact mechanism by which DHA is exported from the peroxisomes either as coenzyme A ester or as free acid has not been deduced sofar.

### (D) bile acid synthesis

**Figure 8:**
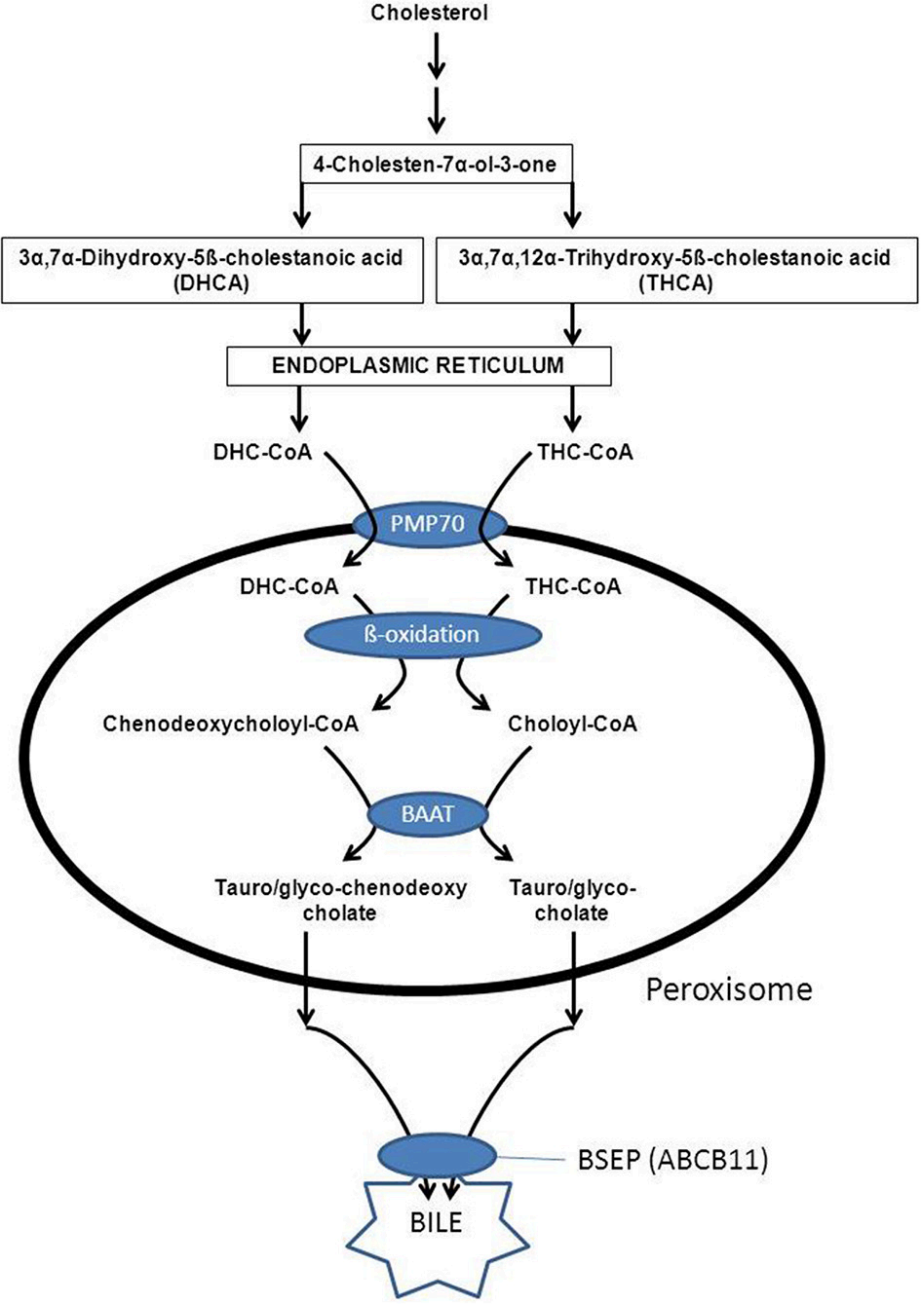
**Schematic diagram showing the unique and important role of peroxisomes in the formation of the primary bile acids cholic acid and deoxycholic acid**. See text for detailed information.

Peroxisomes also play an indispensable role in the biosynthesis of the primary bile acids cholic acid and chenodeoxycholic acid. The underlying basis for the obligatory role of peroxisomes in bile acid formation, resides in the fact that the two bile acid intermediates, i.e., 3alpha, 7alpha-dihydroxy-5beta-cholestanoic acid (DHCA) and 3alpha, 7alpha, 12alpha-trihydroxy-5beta-cholestanoic acid (THCA) undergo beta-oxidative chain shortening in peroxisomes with propionyl-CoA as one product and chenodeoxycholoyl-CoA and choloyl-CoA as the respective other products of beta-oxidation (see Figure 8). The enzymes catalyzing the formation of DHCA and THCA respectively are localized in different subcellular compartments including the cytosol, endoplasmic reticulum, and mitochondrion (CYP27A1) (see Russell, 2003 for review). Activation of DHCA and THCA produced by the mitochondrial enzyme CYP27A1 most likely occurs by the ER enzyme bile acid-CoA ligase (BACL) encoded by SLC27A5 (Wheeler et al., 1997; Falany et al., 2002) after which the two CoA-esters of DHCA and THCA enter the peroxisome. The recent identification of PMP70 deficiency in a patient with markedly elevated DHCA and THCA levels in plasma supported by additional studies in the *Pmp70*(-/-/) mouse has led to the conclusion that the peroxisomal half-ABC-transporter PMP70 (ABCD3) catalyzes the import of these acyl-CoAs into peroxisomes (Ferdinandusse et al., 2015). The actual beta-oxidation of DHC-CoA and THC-CoA is catalyzed by the enzymes ACOX2, D-bifunctional protein, and peroxisomal thiolase-2 (SCPx) (see Figure 4). Subsequently, the two acyl-CoAs are converted into the corresponding taurine and/or glycine esters by the enzyme bile acid-CoA: amino acid *N*-acyltransferase (BAAT). Work by Faber and coworkers has shown that BAAT is a strictly peroxisomal enzyme indicating that tauro/glycocholate and tauro/glycochenodeoxycholate are the true end products of peroxisomal bile acid metabolism (Pellicoro et al., 2007). The tauro/glycoconjugates of cholic acid and chenodeoxycholic acid are then exported from the peroxisome interior via some unknown mechanism into the cytosol followed by the rapid excretion from the hepatocytes into bile via BSEP (ABCB11) localized in the canulicular membrane (Figure 8).

### (E) phytanic acid alpha-oxidation

**Figure 9:**
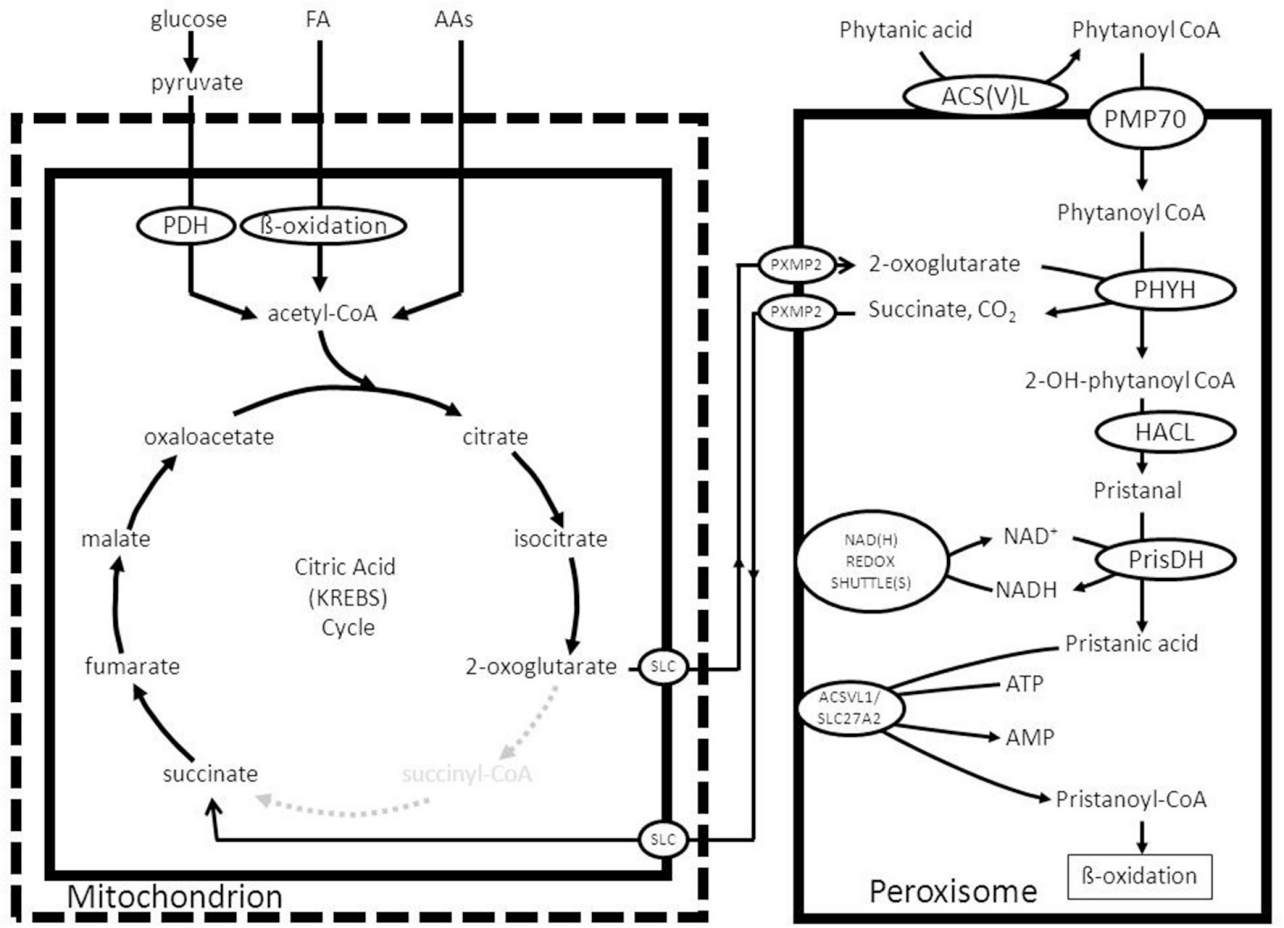
**Functional interplay between mitochondria and peroxisomes in the alpha-oxidation of phytanic acid**. See text for detailed information.

3-methyl-FAs cannot be beta-oxidized right away simply because the methyl-group at the 3-position prohibits beta-oxidation. In order to allow oxidation of 3-methyl-FAs, these FAs first need to undergo one cycle of alpha-oxidation thereby converting the 3-methyl-FA into a 2-methyl-FA which can then be beta-oxidized (Wanders et al., 2011b). Alternatively, 3-methyl-FAs may be oxidized via the omega end so that phytanic acid is actually chain-shortened from the omega-end (see Wanders et al., 2011a for review). The best known FA undergoing alpha-oxidation, is phytanic acid (3,7,11,14-tetramethylhexadecanoic acid) as concluded from observations on a rare disease called Refsum disease in which alpha-oxidation is blocked due to a genetic deficiency of the enzyme phytanoyl-CoA 2-hydroxylase encoded by *PHYH*. Phytanic acid is strictly derived from dietary sources and cannot be synthesized *de novo*. Although not well-studied, the general notion is that phytanic acid is transported throughout the body via the blood in its free as well as esterified form. Indeed, in plasma phytanic acid has been identified in triglycerides but also in other lipid species. According to Wierzbicki et al. (1999) most of the phytanic acid in Refsum disease patients is present in the LDL-fraction. Hydrolysis of LDL-particles after receptor mediated uptake into cells within lysosomes would then release the phytanic acid into the cytosol. The fact that there are multiple acyl-CoA synthetases able to convert phytanic acid into phytanoyl-CoA (see Wanders et al., 2011b for review) would ensure rapid formation of phytanoyl-CoA in the extra-peroxisomal space. This implies that phytanoyl-CoA is the most likely substrate to be transported across the peroxisomal membrane. Based on our own recent work in a patient with a genetic defect in *ABCD3* coding for PMP70 as well as studies in a mutant *Abcd3(-/-*) mouse model we conclude that PMP70 (ABCD3) catalyzes the uptake of phytanoyl-CoA into peroxisomes (Ferdinandusse et al., 2015; see Figure 9).

Once inside the peroxisome interior, phytanoyl-CoA is hydroxylated by the enzyme phytanoyl-CoA 2-hydroxylase first identified by Mihalik et al. (1995) to produce 2-hydroxy-phytanoyl-CoA. The enzyme involved belongs to the group of 2-oxoglutarate-dependent dioxygenases and the hydroxylation of the substrate is driven by 2-oxoglutarate and molecular oxygen with succinate and CO_2_ as products (Mukherji et al., 2003). Subsequently, the enzyme 2-hydroxyacyl-CoA lyase (HACL) cleaves 2-hydroxyphytanoyl-CoA, and a range of other 2-hydroxy acyl-CoAs in fact, between the first and second carbon atom to produce formyl-CoA plus the aldehyde pristanal in case of phytanic acid alpha-oxidation (Foulon et al., 2005). This aldehyde is then converted into the corresponding acid (pristanic acid). Available evidence holds that peroxisomes do contain aldehyde dehydrogenase activity as shown for pristanal by Jansen et al. (2001). The true identity of this enzyme activity has not been settled definitively. For humans it has been suggested that FALDH-V, a truncated splice product produced from the *ALDH3A2* gene, is directed to peroxisomes (Ashibe et al., 2007). The bulk of FALDH-activity produced from the *ALDH3A2* gene, however, is located in the endoplasmic reticulum (ER). Whether FALDH-V is truly the enzyme responsible for the pristanal dehydrogenase activity in peroxisomes remains doubtful, however, for various reasons including the fact that FALDH-V appears to be a membrane-bound enzyme with its catalytic domain exposed to the cytosol, whereas the peroxisomal pristanal dehydrogenase activity is catalyzed by a soluble matrix enzyme (Jansen et al., 2001). Furthermore, phytanic acid alpha oxidation is fully normal in patients suffering from Sjögren Larsson syndrome (SLS) caused by mutations in the *ALDH3A2* gene. In line with these results, phytanic acid does not accumulate in SLS-patients. Figure 9 depicts the organization of the alpha-oxidation system in peroxisomes as envisaged right now with phytanoyl-CoA 2-hydroxylase, HACL, and also the putative pristanal dehydrogenase all localized in the matrix of the peroxisome. The alpha-oxidation machinery can only work efficiently if the NADH produced in the aldehyde dehydrogenase reaction is reoxidized back to NAD^+^ as described above. Furthermore, constant supply of 2-oxoglutarate is required, coupled to the removal of succinate. Both 2-oxoglutarate and succinate can probably move freely through the peroxisomal membrane via the peroxisomal porine PXMP2. Since succinate is a 4-carbon molecule, whereas 2-oxoglutarate has 5-carbon atoms, reconversion of succinate back into 2-oxoglutarate can only be achieved via one of the carboxylases or some other mechanism. A possible role for one of the four known carboxylases, including pyruvate carboxylase, acetyl-CoA carboxylase, propionyl-CoA carboxylase, and methylmalonyl-CoA carboxylase is hard to envisage. However, conversion of succinate back into 2-oxoglutarate can also occur in the mitochondrion by using part of the citric acid cycle and in particular the citrate synthase reaction which can turn a C4-molecule like oxaloacetate into the 6-carbon molecule citrate. The mechanism would then be that succinate enters the mitochondrion via the mitochondrial dicarboxylate carrier and is converted back into 2-oxoglutarate via the concerted action of succinate dehydrogenase, fumarase, malate dehydrogenase, citrate synthase, and NAD-linked isocitrate dehydrogenase followed by export of 2-oxoglutarate via the mitochondrial carrier specific for 2-oxoglutarate (see Figure 9).

With respect to one of the other products of alpha-oxidation, i.e., formyl-CoA, the current notion holds that formyl-CoA is rapidly hydrolyzed spontaneously to produce free CoASH and formic acid (Croes et al., 1997). Formic acid can be degraded via two pathways including: (1.) a catalase mediated pathway in which catalase operates in the peroxidative mode and (2.) via the folate-dependent pathway (Tephly, 1991). Finally, the CoA released from formyl-CoA could be used to convert pristanic acid to pristanoyl-CoA as described above. In fact, peroxisomes do contain pristanoyl-CoA synthetase activity which is probably catalyzed by the enzyme ACSVL1 (SLC27A2) as already mentioned above. Figure 9 shows the final scheme in which the considerations above have been incorporated and used to construct a feasible model.

### (F) glyoxylate detoxification

**Figure 10:**
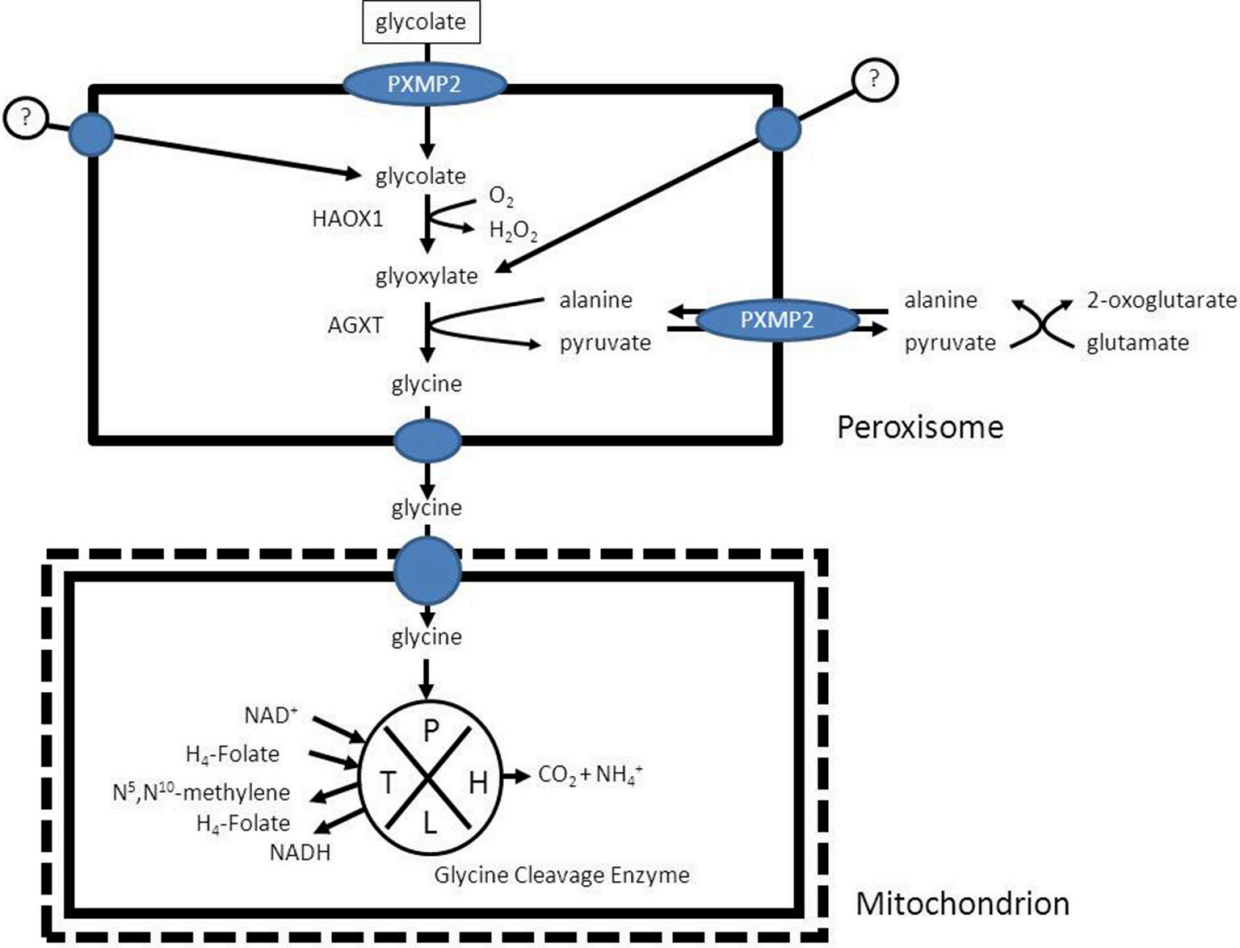
**The detoxification of glyoxylate in peroxisomes as catalyzed by the enzyme alanine glyoxylate aminotransferase (AGXT)**.

In humans the enzyme alanine glyoxylate aminotransferase (AGXT) is the principal enzyme involved in the detoxification of glyoxylate, is strictly peroxisomal in human liver (Danpure and Jennings, 1986) and a deficiency of this enzyme causes hyperoxaluria type 1 (Danpure et al., 1987) which in its extreme form may be lethal due to the accumulation of calcium oxalate in multiple tissues including the kidneys, liver, and heart (see Salido et al., 2012 for review). The product of the AGXT reaction in peroxisomes is pyruvate which needs to be reconverted into alanine via different transaminases localized in the cytosol or degraded in the mitochondrion via the enzyme pyruvate dehydrogenase, which again shows the interaction of peroxisomes with multiple subcellular compartments including the cytosol and the mitochondrion in the case of glyoxylate metabolism (Figure 10; Salido et al., 2012). Glycine is further metabolized in mitochondria and broken down via the glycine cleavage enzyme which is made up of four different proteins, named P-, T-, H-, and L-protein (see Figure 10; Kikuchi et al., 2008 for review).

It should be noted that much remains to be learned about the metabolic precursors of glyoxylate although glycolate is definitely one of the major sources of glyoxylate with the peroxisomal enzyme 2-hydroxy acid oxidase (HAO1; alternative named: glycolate oxidase) as the enzyme responsible for the conversion of glycolate to glyoxylate (Vignaud et al., 2007). Other known sources of glyoxylate are hydroxyproline (Knight et al., 2006) and glycine.

### (G) amino acid metabolism

Peroxisomes also play an indispensable role in the degradation of a range of amino acids, notably the D-amino acids. Indeed, mammalian tissues contain at least two different degradative enzymes that are stereospecific for D-amino acids including D-amino acid oxidase (DAO; also known as DAAO) and D-aspartate oxidase (DDO; also known as DASPO). Both DAO and DDO are FAD-linked flavoproteins able to catalyze the oxidative deamination of D-amino acids to produce H_2_O_2_, ammonia, and the corresponding 2-oxoacid. The *DAO* gene product displays a broad substrate specificity and reacts with a range of neutral and basic D-amino acids including D-serine, D-alanine, and others (Krebs, 1935; Dixon and Kleppe, 1965). The other oxidase (DDO) is highly specific for acidic D-amino acids such as D-aspartate and D-glutamate but also reacts with N-methyl-D-aspartate acid. Mammalian DAO and DDO are presumed to regulate the levels of several endogenous and exogenous D-amino acids including D-serine and D-aspartate in various organs notably the brain. D-serine for instance binds to the glycine binding site of the N-methyl-D-aspartate (NMDA) receptor and potentiates glutamatergic neurotransmission in the central nervous system. Several lines of evidence suggest that D-serine plays an important role in the regulation of brain functions by acting as co-agonist for the NMDA receptor and perturbations in D-serine in the nervous system have recently been implicated in the pathophysiology of various neuropsychiatric disorders (see Katane et al., 2015 for references). Recent studies have also shown that D-aspartate acts as signaling molecule in nervous and neuroendocrine systems at least in part by binding to the NMDA receptor and, thus plays an important role in the regulation of brain function (Katane and Homma, 2011; Errico et al., 2012; Ota et al., 2012). Furthermore, peroxisomes, at least in humans, are the sole site of L-pipecolic acid oxidase activity which is a metabolite derived from lysine (Wanders et al., 1988; Mihalik et al., 1989). There is currently very little information in literature on the functional interplay between peroxisomes and other subcellular compartments in the oxidation of the various amino acids in humans is concerned.

### (H) ROS/RNS-metabolism

In line with its name, the peroxisome also plays a major role in cellular ROS/RNS-metabolism. Indeed, peroxisomes contain a large number of ROS-producing enzymes of which the acyl-CoA oxidases are the most abundant being present in virtually all peroxisomes independent of the tissue and cell type involved. Other H_2_O_2_ producing oxidases include D-amino acid oxidase (DAO), D-aspartate oxidase (DDO), L-pipecolate oxidase (PIPOX), 2-hydroxy acid oxidases (HAO), polyamine oxidase, and xanthine oxidase. Furthermore, the inducible form of NOS (NOS2) is localized in peroxisomes (for review see Schrader and Fahimi, 2006; Antonenkov et al., 2010; Fransen et al., 2012; Nordgren and Fransen, 2014; Lismont et al., 2015). In addition, peroxisomes contain a large network of enzymatic and also non-enzymatic antioxidants that protect the organelle from oxidative damage. The main antioxidant enzymes include thioredoxin 2(TRX2), thioredoxin reductase (TXNRD2), the glutaredoxins 2 (GLRX2), and 5 (GLRX5), the peroxiredoxins 3 (PRDX3), and 5 (PRDX5), GSH peroxidase 1 (GPX1), oxidized glutathione (GSSG) reductase (GSR), and the copper/zinc (SOD1)- and manganese (SOD2)-containing SODs (see Lismont et al., 2015 for review). Recent work by Fransen and coworkers (Wang et al., 2013) has shown that also with respect to ROS/RNS-metabolism there is marked functional interplay between peroxisomes and other subcellular organelles, notably mitochondria (Wang et al., 2013).

## Concluding remarks

Peroxisomes play a crucial role in cellular metabolism as exemplified by the different inborn errors of metabolism caused by a deficiency of one of the peroxisomal enzymes (Table 1) as reviewed in this paper. It is also fully clear that the metabolic capabilities of peroxisomes are very much dependent on the functional interplay with other organelles, notably the mitochondrion and endoplasmic reticulum. Although much has been learned about the functional organization of the peroxisome in terms of the enzymes involved and the end-products of peroxisomal metabolism, there are still substantial gaps in our knowledge about peroxisome metabolism. These include the question how substrates and products of peroxisome metabolism are transported across the peroxisomal membrane, especially since PXMP2 only allows passage of small molecules with an M_*w*_ < 300 (Antonenkov and Hiltunen, 2012). One other area which has remained relatively unexplored involves the mechanism of transfer of the end products of peroxisome metabolism from the peroxisome to other organelles like the mitochondrion and ER. It is gratifying to see that several recent reports are beginning to shed light on the mechanisms involved in the physical interaction between individual organelles and the proteins involved. This is especially true for the peroxisome-mitochondrion association with the identification of PEX11 and its interaction with the ERMES complex (Mattiazzi Usaj et al., 2015; see Schrader et al., 2015 for review).

### Conflict of interest statement

The authors declare that the research was conducted in the absence of any commercial or financial relationships that could be construed as a potential conflict of interest.

## Abbreviations

ACAA1: peroxisomal 3-ketoacyl-CoA-thiolase1
ACBP: acyl-CoA binding protein
ACOX: acyl-CoA oxidase
ADHAPS: alkyl-dihydroxyacetonephosphate synthase
AGPS: alkyl-glyceronephosphate synthase
AMACR: 2-methyl-acyl-CoA racemase
BAAT: bile acid-CoA: amino acid *N*-acyltransferase
BSEP: bile salt export pump
CACT: carnitine/acylcarnitine translocase
CrAT: carnitineacetyltransferase
CrOT: carnitine octanoyltransferase
DHA: docosahexaenoic acid
DHAP: dihydroxyacetone phosphate
DHAPAT: dihydroxyacetonephosphate acyltransferase
DHCA: dihydroxycholestanoic acid
EPL: etherphospholipid
ER: endoplasmic reticulum
ETF: Electron-Transfer-Flavoprotein
FA: fatty acid
FAR1: fatty acyl-CoA reductase 1
FAR2: fatty acyl-CoA reductase 2
G3PDH, GPD1: glycerol-3-phosphate dehydrogenase
G3P: glycerol-3-phosphate
GNPAT: glycerone-phosphate O-acyltransferase
IDH: isocitrate dehydrogenase
MUFA/PUFA: mono/poly-unsaturated FA
PMP70: peroxisomal membrane protein 70
ROS: reactive oxygen species
RNS: reactive nitrogen species
THCA: trihydroxycholestanoic acid
VLCFA: very long-chain fatty acid
ZS: Zellweger syndrome.
